# Draft genome sequence of biofilm-forming *Enterococcus faecalis* BAU_Ef01 strain isolated from shrimp (*Penaeus indicus*) in Bangladesh

**DOI:** 10.1128/MRA.00551-23

**Published:** 2023-08-18

**Authors:** Md. Ashek Ullah, Md. Saiful Islam, Md. Liton Rana, Farhana Binte Ferdous, Fahim Haque Neloy, Sadia Afrin Punom, Jayedul Hassan, Md. Tanvir Rahman

**Affiliations:** 1 Department of Microbiology and Hygiene, Faculty of Veterinary Science, Bangladesh Agricultural University, Mymensingh, Bangladesh; DOE Joint Genome Institute, Berkeley, California, USA

**Keywords:** white shrimp, *E. faecalis*, whole-genome sequencing, biofilm, virulence, antimicrobial resistance, Bangladesh

## Abstract

Here, we sequence and analyze a biofilm-forming strain of *Enterococcus faecalis* BAU_Ef01 isolated from a shrimp in Bangladesh. The whole genome of the strain had a length of 2,862,301 bp, 38 contigs, an average G+C content of 37.36%, 80.0× genome coverage, and 35 predicted antibiotic resistance and virulence genes each.

## ANNOUNCEMENT

Environmental ecosystems, especially seafood, contain various commensal microorganisms, including *Enterococcus faecalis*, that can be transferred to humans through the food chain. *E. faecalis* is well known for forming biofilms to endure its challenging surrounding conditions (1). Biofilm-forming bacteria possess several advantages over single planktonic cells, such as enhanced resilience against environmental stress conditions, sanitizers, and antimicrobial agents (2). Moreover, *E. faecalis* strains with high virulence and resistance are thought to be found in seafood samples (3). *E. faecalis* is one of the primary triggers of hospital-acquired infections and can cause serious infections, including meningitis, bacteremia, urinary tract infections, gastrointestinal infections, periodontitis, and others (4).

From October 2021 to March 2023, white shrimp (*Penaeus indicus*) samples were collected from Cox’s Bazar (21.4272°N, 92.0058°E) and transferred to our laboratory (21.4272°N, 92.0058°E). Subsequently, samples were processed following the previously described procedure (5). Each shrimp sample’s brain, leg, muscle, and intestine were blended and then used for enrichment in a nutrient broth (HiMedia, India), streaked on Enterococcus agar (HiMedia, India) media, and incubated aerobically again overnight at 37°C. Then suspected pure colonies were subjected to Gram staining and biochemical tests to isolate *E. faecalis* (6). Finally, *E. faecalis* was identified through a matrix-assisted laser desorption ionization–time of flight mass spectrometry assay (7). The biofilm-forming ability of the isolate was evaluated using the Congo Red test (8). A strong biofilm-forming *E. faecalis* isolate, showing dry filamentous crusty black colonies on the Congo Red agar plate (9), was selected for the present study and incubated overnight in nutrient broth (HiMedia, India) at 37°C. The DNA was then extracted from the collected broth culture utilizing a DNA mini kit (QIAGEN, Hilden, Germany). A NanoDrop 2000 UV-Vis Spectrophotometer (Thermo Fisher, Waltham, MA, USA) was employed to determine the DNA concentration and assess its purity. Following the manufacturer’s guidelines, the Nextera DNA Flex Library Prep Kit (Illumina, San Diego, CA, USA) was used to create the DNA library. Genome sequencing was performed on the Illumina NextSeq 2,000 platform, generating paired-end reads with a length of 2 × 150 base pairs (bp). The genome assembly was carried out using Unicycler (10), preceded by trimming the raw paired-end reads using Trimmomatic (11). Subsequently, FastQC (12) was utilized to evaluate the quality of the trimmed reads. The genome was annotated in Prokka (13), PATRIC (14), and PGAP (15). The antibiotic resistance genes (ARGs) were determined using the CARD (16), NDARO (17), ResFinder (18), and PATRIC (14), and the virulence factor genes (VFGs) were ascertained by utilizing VFDB (19), VirulenceFinder (20), PATRIC_VF (21), and Victors (22). We employed PathogenFinder (23) for identifying the pathogenicity index, MLST (24) for the sequence type, and RAST (25) for the metabolic functional features in our assembled genome. Here, all tools were run with default parameters unless otherwise specified.

The assembled draft *E. faecalis* BAU_Ef01 genome consisted of 38 contigs (four L50 contigs with an N50 value of 326,766 bp), with a cumulative length of 2,862,301 bp and an average G+C content of 37.36%. In the PATRIC annotation, the genome contained 2,728 protein-coding sequences, 48 transfer RNA genes, and three ribosomal RNA genes. This genome corresponds to sequence type ST862 based on MLST and exhibits a probability of 89.6% for being a human pathogen based on PathogenFinder. The *E. faecalis* BAU_EF01 genome had 35 ARGs under different antimicrobial classes, including glycopeptides, tetracyclines, macrolides, fluoroquinolones, rifamycins, diaminopyrimidine, lincosamides, streptogramins, etc. Our assembled genome harbored 35 predicted VFGs under various virulence factors, including biofilm formation, toxin, adherence, antiphagocytosis, enzyme, immune evasion, and others. Moreover, our genome contained 779 subsystems with 29% coverage and harbored 1,075 genes for different subsystem features, such as virulence, disease, and defense, carbohydrates, amino acids and derivatives, nucleosides and nucleotides, cofactors, vitamins, prosthetic groups, pigments, iron acquisition and metabolism, etc.

## Data Availability

The WGS shotgun analysis of *E. faecalis* BAU_Ef01 was submitted to GenBank with the assigned accession number JAQSHP000000000. The associated data, including the raw reads, were deposited with BioProject accession number PRJNA932982, BioSample accession number SAMN33217485, and SRA accession number SRR23380394. In this paper, the specific version being referred to is identified as JAQSHP000000000.1.
